# Harnessing the potential of millets for climate-resilient and sustainable agriculture

**DOI:** 10.3389/fpls.2025.1574699

**Published:** 2025-06-11

**Authors:** Bishal Mukherjee, Ratnesh Kumar Jha, Abdus Sattar, Suman Dutta, Urjashi Bhattacharya, Snigdha Samanta, Boris Huirem, Shivam Kumar Singh, Suddhasuchi Das, Santanu Kumar Bal

**Affiliations:** ^1^ Centre for Advanced Studies on Climate Change, Dr. Rajendra Prasad Central Agricultural University, Pusa, Bihar, India; ^2^ Department of Genetics and Plant Breeding, School of Agriculture and Rural Development, Ramakrishna Mission Vivekananda Educational and Research Institute, Belur Math, Howrah, West Bengal, India; ^3^ Department of Agronomy, School of Agriculture and Allied Sciences, The Neotia University, Sarisa, West Bengal, India; ^4^ Department of Agricultural Entomology, School of Smart Agriculture, Adamas University, Barasat, West Bengal, India; ^5^ Department of Food Science, School of Agriculture and Allied Sciences, The Neotia University, Sarisa, West Bengal, India; ^6^ Malda Krishi Vigyan Kendra, Uttar Banga Krishi Viswavidyalaya, Ratua, Malda, West Bengal, India; ^7^ ICAR-Central Research Institute for Dryland Agriculture, Hyderabad, India

**Keywords:** climate-resilient, crop alternative, dietary nutrients, food security, millets, underutilized crops

## Abstract

Millets are increasingly gaining global attention for their immense potential to address major challenges such as hunger and malnutrition, and the climatic risks on agricultural production. With the world’s population steadily increasing, it is essential to find sustainable solutions for regional food and nutrition security, and support the livelihoods of farmers—especially under changing climate. Among various crops, small millets offer a promising yet underutilized option in the pursuit of food and nutritional security. These crops possess superior nutritional profiles compared to traditional staple cereals and demonstrate exceptional resilience to harsh environmental conditions. They can grow with minimal irrigation and thrive on poor-quality soils. This makes millets ideal for sustainable agriculture in challenging environments. This review highlights the current status of small millets, including their nutritional and health benefits, processing techniques, and recent advancements in genomics. It emphasizes the importance of integrating small millets into mainstream agricultural systems and applying omics technologies to fully realize their potential in combating food insecurity under climate stress. Additionally, the review explores modern genomic and genetic engineering approaches that aim to enhance the climate resilience of small millets. By understanding the molecular basis of these traits, researchers can develop improved varieties with greater adaptability and consistent yields under variable environmental conditions. The comprehensive analysis presented in this review serves as a strategic roadmap for leveraging next-generation technologies to improve key traits in small millets. The ultimate goal is to develop superior varieties that can address the complex challenges of agri-food production system under climate uncertainties. Furthermore, the review outlines pathways to increase the commercial appeal and market viability of millets. By focusing on the development and promotion of small millets, the agricultural sector can take significant steps toward achieving food, nutritional, and economic security in the wake of growing global challenges.

## 1 Introduction

Diversity of crops is pivotal for sustainable agriculture and ensuring global food and nutritional security amid climate change. In this context, millets, often referred to as “Nutri-cereals,” emerge as a promising alternative to conventional staple crops. They are rich sources of essential macronutrients, micronutrients, dietary fibers, essential amino acids, minerals, antioxidants, trace elements, proteins, lipids, and carbohydrates (Banerjee et al., 2020). Millets flourish across a wide range of climatic and soil conditions particularly in marginal and degraded lands, exhibiting remarkable resilience to varieties of environmental stresses (Sharma et al., 2021; Maity et al., 2023). Their ability to thrive under challenging conditions with minimal water and fertility requirements, makes them an invaluable resource for sustainable agriculture especially in drought-prone areas and soils affected by acidity, salinity or aluminum toxicity (Raj et al., 2024). Due to their climate resilient characteristics and superior nutrient profile, millets are aptly termed as “miracle grains” (Srivastava and Arya, 2021). Despite these benefits, millets remain marginalized in global agriculture, often labeled as “orphan cereal crops” due to the dominance of major cereals such as wheat, rice and maize (Muthamilarasan and Prasad, 2021). As global food security and nutritional deficiencies present mounting challenges for a growing human population, crop diversification becomes critical for securing food availability (Srivastava and Arya, 2021). However, shifting climate patterns threaten food security by adversely impacting agricultural yields (Oluwole et al., 2021). The prevalence of malnutrition, or “hidden hunger,” particularly in developing nations, is largely driven by the reliance on calorie-dense monocultures that lack essential micronutrients. Staple crops like wheat, rice, and maize often require substantial inputs of fertilizers and herbicides to sustain yields (Kreitzman et al., 2020; Mukherjee et al., 2023). In regions such as Africa and Asia, limited dietary diversity contributes significantly to widespread nutritional deficiencies (Wakeel et al., 2018). Incorporating millets into cropping system offers a powerful approach for improving dietary diversity and enhancing resilience to biotic and abiotic stresses, thereby contributing to food security and providing economic stability for farmers (Mustafa et al., 2021). Although millets are often termed as ‘minor crops’ due to their limited acreage (Yenagi et al., 2010), they possess immense nutritional and medicinal values. Millets are C_4_ plants characterized by high levels of photosynthetic efficiency, a short lifespan, superior dry matter production, and exceptional heat and drought tolerance. Consequently, they offer more consistent yields compared to other staple crops in low rainfall locations (Tadele, 2016). Moreover, research on small millets has identified numerous candidate genes associated with yield improvements, stress resilience and other vital agronomic traits (Lata and Shivhare, 2017; Wambi et al., 2021).

Recent studies have illuminated the phenotypic diversity, climate resilience traits, genome-wide markers, and the heritability of key attributes in small millets. In this context, the review examines recent advancements in next-generation technologies and explore how they can be used to enhance small millet production sustainably.

## 2 Global scenario of millets cultivation

Millets are thought to be among the ancient cultivated crops of the world and have been found to be staple food material in parts of Central and Eastern Asia, European countries, Russia, China, India, and parts of Africa (Baltensperger, 1996). Most of the millets originated from Africa and later domesticated in other parts of the world. Globally millets (pearl millet and minor millets) are cultivated in more than 93 countries out of which only 7 countries constitute more than 1 M ha area under millets whereas 25 countries have more than 0.1 M ha harvested area (FAOSTAT, 2018).

According to FAOSTAT (2021), the global millet production in 2019-20 was 84.17 million metric tons from an area of 70.75 million hectares, of which 20.50% is produced in India. Presently, millets are used in the diets of about 90 million people in Asia and Africa. Africa accounts for more than 55 percent of global production, followed by Asia, which accounts for almost 40 percent, while Europe accounts for around 3 percent of the global market (Prashanthi and Reddy, 2023). Among different regions of Africa, West Africa recorded the highest millet cropping area with 14.3 M ha followed by North Africa (3 M ha). West Africa alone contributed around 44.3% of the world millet growing area (FAOSTAT, 2018). Among the top millet growing countries of the world, India ranked first with 15.29 M ha harvested area followed by Niger (7.03 M ha), Sudan (3.75 M ha), Nigeria (2.7 M ha), Mali (2.15 M ha), Burkina Faso (1.39 M ha), and Chad (1.22 M ha). India is the largest grower of millets and contributes about 26.6% of the global harvested area (FAOSTAT, 2018). United States (0.16 M ha) followed by Argentina are the main millet growers of America. Ukraine, Poland, France, and Belarus are the largest millet growing countries in Europe. Over the span of 58 years from TE 1963 to TE 2021 (**Table 1**), there has been a decrease in the worldwide cultivation area of millets by 29%, i.e., from 43.40 million hectares to 30.88 million hectares. Although America and Oceania have consistently sustained area under millets cultivation, Asia and Europe have experienced a stagnation. Though the area under cultivation had a declining trend, still Asia continues to be the largest contributor to the global share of millets production (52.74%), observing a modest increase of 2.55%, reaching 15.68 million tons (FAOSTAT, 2023).

**Table 1:** Trends in Millet area, production and productivity scenario of the world.

Sl No.	Region	Area (M ha^*^)		Production (mt^#^)		Productivity (kg ha^-1^)	
		TE 1963	TE 2021	TE 1963	TE 2021	TE 1963	TE 2021
1.	Africa	11.84	19.09	6.94	13.06	586.13	683.47
2.	America	0.27	0.22	0.33	0.32	1222.57	1457.60
3.	Asia	27.17	11.07	15.29	15.68	562.40	1414.23
4.	Europe	4.09	0.45	2.38	0.63	582.70	1416.00
5.	Oceania	0.03	0.04	0.03	0.04	1086.97	1020.90
6.	World	43.40	30.88	24.97	29.73	575.37	962.60

*, million hectors; #, million tons. Source: FAOSTAT (2023). TE, Time Estimate.

Among the different categories of millets, sorghum, and pearl millet accounts for 92.6% of the global millets production. Out of this, sorghum is the major millet accounting for 65.8% of the total production of millets around the world (Prashanthi and Reddy, 2023). Finger millet, foxtail millet, proso millet, little millet, and kodo millet are the other millets which altogether account for 7.94% of global millet production. Even though India produces most millets in the world, more than 40% of millet is consumed in African countries, especially Niger, Mali, Nigeria, Burkina Faso, and Sudan. In India, millet production is concentrated mainly in dry and arid regions where low and erratic rainfall patterns are observed. Pearl millet is the most widely produced millet in India, which is mainly grown in Rajasthan, Uttar Pradesh, Gujrat, Madhya Pradesh, and Haryana accounting for 56% of the total millets production of the country (INDIASTAT, 2020). Among the minor millets, finger millet is the most widely produced millet in India with 1.79 Mt of production from the total cropped area of 1.17 M ha (INDIASTAT, 2020). Rajasthan leads the millets production of the country followed by Karnataka, Madhya Pradesh, Uttar Pradesh, Tamilnadu, and Telangana (Agriculture Statistics at a glance, 2021). A brief scenario of millets production in different states of India is given in **Table 2**.

**Table 2:** Millets production scenario in India.

Sl No.	Region	2018-19		2019-20		2021-22	
		Area (M ha^*^)	Production (mt^#^)	Area (M ha)	Production (mt)	Area (M ha)	Production (mt)
1.	Rajasthan	5.83	6.99	6.13	7.29	3.90	4.41
2.	Karnataka	3.01	5.52	3.13	6.45	1.65	1.89
3.	Madhya Pradesh	1.84	5.15	1.84	4.82	0.85	1.12
4.	Maharashtra	3.32	3.10	4.33	4.73	2.35	2.12
5.	Uttar Pradesh	1.91	3.95	1.99	4.47	1.10	1.98
6.	Tamil Nadu	0.92	3.71	0.96	3.33	0.52	1.00
7.	Telangana	0.61	2.16	0.66	3.14	0.35	0.65
8.	Andhra Pradesh	0.49	1.86	0.56	2.68	0.40	0.75

*, million hectors; #, million tons. Source: Agriculture Statistics at a glance, 2021

Agriculture Statistics at a glance, 2023

## 3 Classification of millets: A brief overview

**Figure 1:**
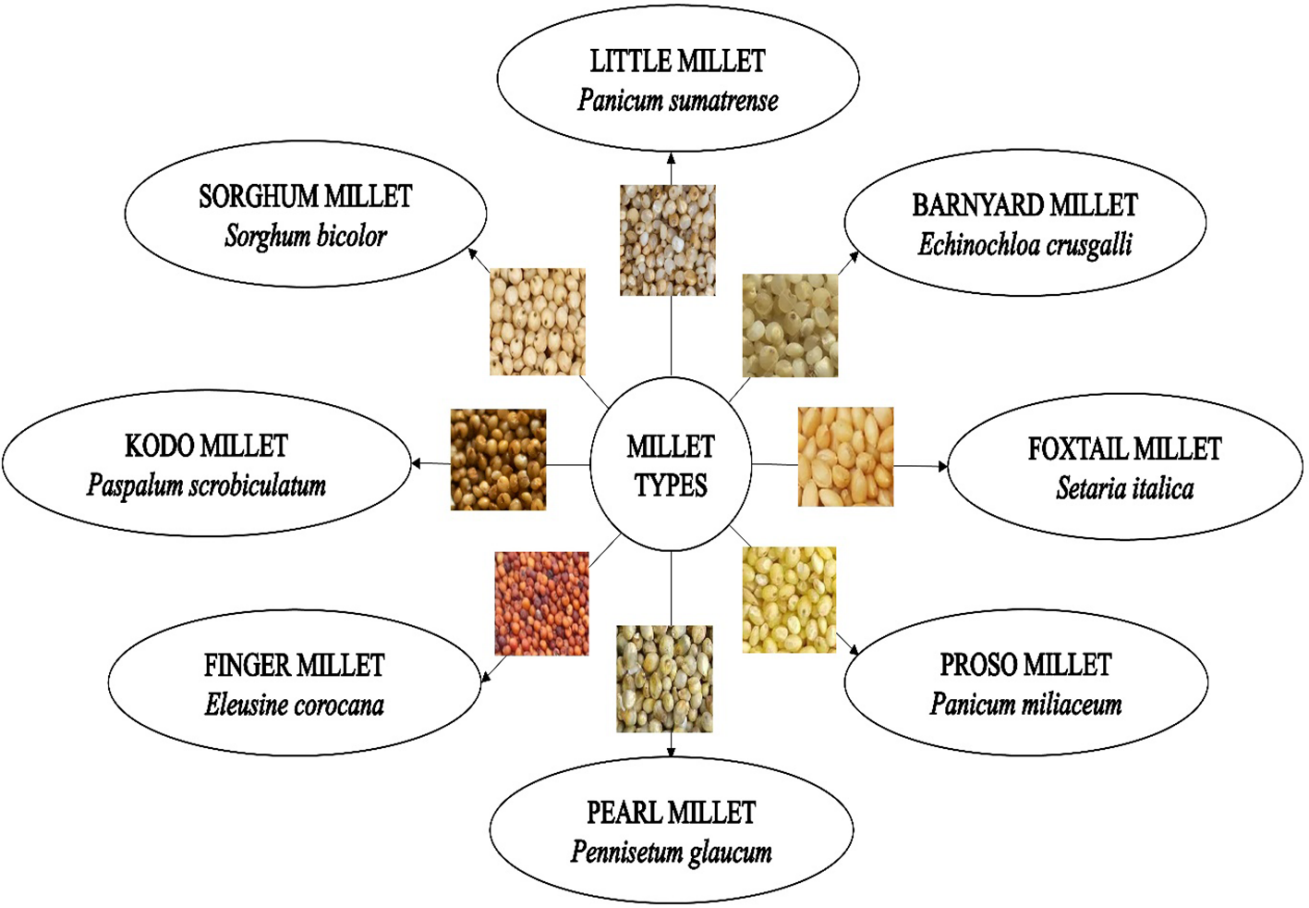
Different types of millets with their botanical name.

There are 14 species of millets classified under 10 different genera, including pearl millet (*Pennisetum glaucum* L.), foxtail millet (*Setaria italica* L. subsp. *italica*), Finger millet (*Eleusine coracana* L.), barnyard millet (*Echinochloa esculenta* A.), proso millet (*Panicum miliaceum* L. subsp. *miliaceum*), kodo millet (*Paspalum scrobiculatum* L.), and little millet (*Panicum sumatrense* Roth.) which are cultivated widely throughout the world. Literature on millets, however, can be very difficult to study since the same species of millets has different common names and vernacular names. Brief details of different types of millets are given in **Table 3**, **Figure 1**.

**Table 3:** Classification of Millets with their brief description.

Sl No.	Name of Millet	Brief description	References
Major Millets			
1.	Sorghum (*Sorghum bicolor* L.)	Known as Jowar/Camel crop, warm season crop, intolerant of low temperatures but fairly resistant to serious pests and diseases. It is rich in protein, fibre, thiamine, riboflavin, folic acid etc.	Dayakar Rao et al. (2017); Harinarayanan et al. (2023); Sharma et al. (2024)
2.	Pearl millet (*Pennisetum glaucum* L.)	Known as Bajra, originated in Central tropical Africa and is widely distributed in the drier tropics and India. It is well adapted to adverse environmental conditions with rainfall less than 250 mm and temperature of above 30°C and mainly grown by subsistence farmers throughout Africa, Asia, and Australia. It contains high proportion of protein (12-16%) and dietary fibre (11.5%) also.	Paschapur et al. (2021); Harinarayanan et al. (2023); Dayakar Rao et al. (2017); Sharma et al. (2024)
3.	Finger millet (*Eleusine coracana* L.)	Known as Ragi/Marwah, originated from highlands of Ethiopia and Uganda. It can thrive under dry and hot conditions upto 35°C and low to moderate rainfall. It contains 6-8% protein, 1.5-2% fat, huge calcium (300-350 mg/100 g) and S rich amino acids. Asia and Africa are major centers of production and India is the leading producer of ragi in the world.	Paschapur et al. (2021); Rao et al. (2017); Harinarayanan et al. (2023)
Minor Millets			
4.	Foxtail millet (*Setaria italica* L.)	Known as Kakum/Kaon, originated from China, one of the ancient cereals cultivated in Europe and Asia, contains high carbohydrate and protein compared to rice, sweety nutty flavored grain. The crop is well adapted to cooler climates and matures in less than 70–120 days.	Harinarayanan et al. (2023); Jiaju and Yuzhi (1994); Sharma et al. (2024)
5.	Kodo millet (*Paspalum scrobiculatum* L.)	Known as Kodon/ditch millet, majorly produced in India, well adapted to tropical and sub-tropical climatic conditions, it takes 120–180 days to mature and the grain yields are very low (250–1000 kg/ha). It contains higher protein (11%), low fat (4.2%), higher dietary fibre (14.3%) in grain.	Paschapur et al. (2021); Sharma et al. (2024)
6.	Barnyard millet (*Echinochloa esculenta* A.)	Known as Sanwa/Japanese millet, originated in Japan province, crop prefers warm climate but can be cultivated under cool climate also. Grains are richest source of crude fibre and protein.	Harinarayanan et al. (2023); House (1995); Dayakar Rao et al. (2017)
7.	Little millet (*Panicum sumatrense* Roth.)	Known as Kutki/Shavan, crop adapted to both dry and humid regions. Grains contain high dietary fibre (38%), iron and antioxidants.	Paschapur et al. (2021); Sharma et al. (2024)
8.	Proso millet (*Panicum miliaceum* L. subsp. *miliaceum*)	Known as Chenna/broom millet, originated from China, well adapted to temperate climatic conditions up to altitudes of 3500 m and various soil types. Grains contain higher protein (12.5%), manganese, carbohydrate and fatty acid.	Baltensperger (2002); Harinarayanan et al. (2023); Dayakar Rao et al. (2017); Sharma et al. (2024)

## 4 Nutrition profile in millets

**Figure 2:**
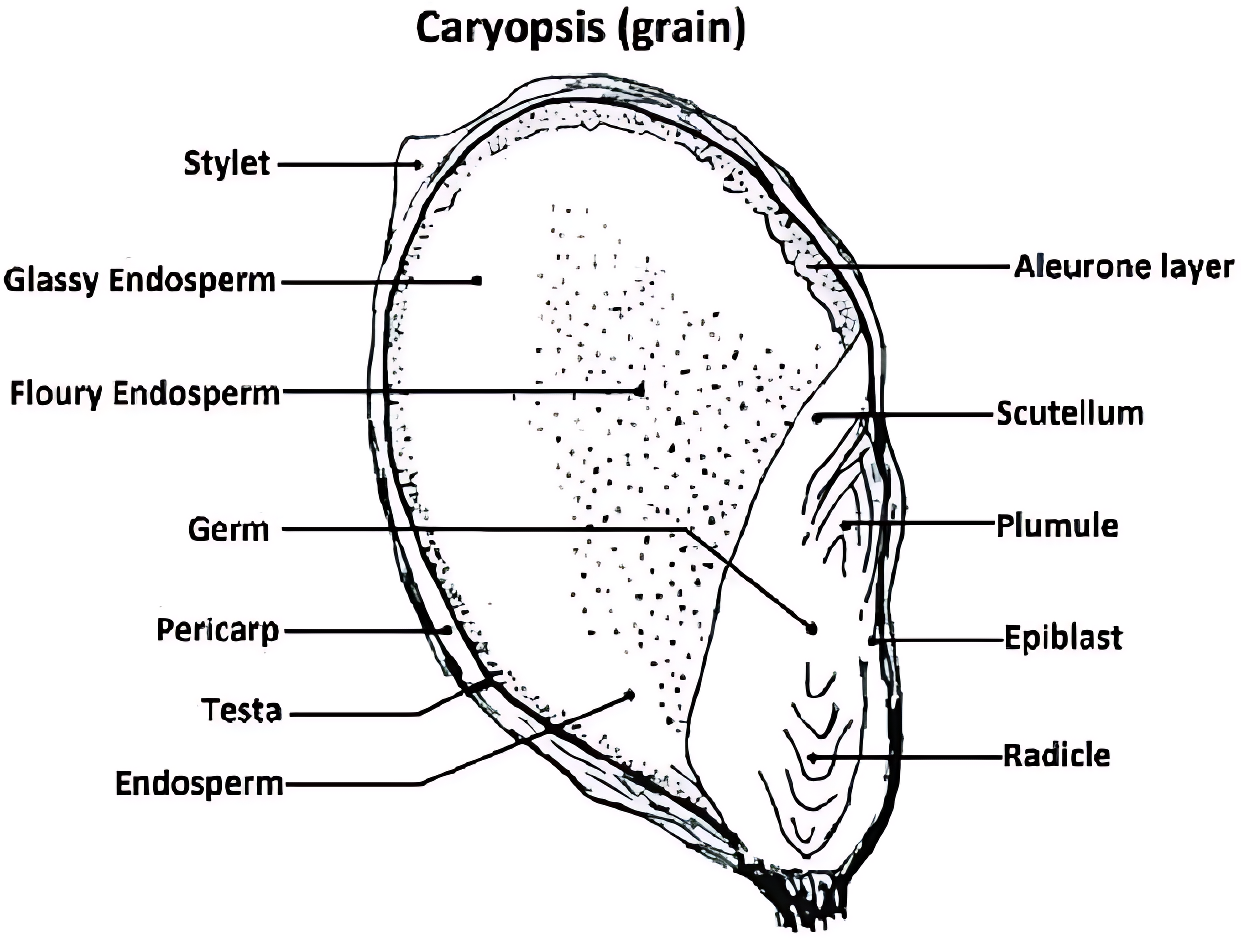
General structure of millet grain (Rao et al., 2017).

Millets are considered as primary food in many places, especially in India and Africa (Meena et al., 2021). Millets, known for their drought resistance amidst environmental adversities, serve as a nutritional powerhouse, offering a rich array of both macro and micronutrients. They are recognized for their nutraceutical benefits, including anti-diabetic and anti-hypertensive properties. Typically, the composition of millet grains includes 65-75% carbohydrates, 6-8% proteins, 1-1.7% fats, 18%-20% dietary fibers, and 2-2.5% minerals, enriched further with essential vitamins and bioactive compounds such as phytates, tannins, and polyphenols (Chethan and Malleshi, 2007; Longvah et al. 2017; Malleshi and Hadimani, 1993). This nutritional profile positions millets as nutritionally superior to major cereals like wheat, rice, and maize. The endosperm of millet grains is notably concentrated in starch, proteins, fats, and a modest amount of fibers, underscoring their value in a balanced diet (Rao et al., 2018). The germ consists of protein, fats and minerals while the peripheral layer comprises dietary fibers, minerals and phenolic compounds (**Figure 2**). Among micronutrients various trace elements, vitamins and carotenoids are the important constituents in millets. Because of wide range of its nutritional composition, millets are termed as nutricereals. Apart from these, it also contains phytochemicals and antinutrients.

### 4.1 Carbohydrates

Millets are rich in carbohydrates, primarily in the form of starch, which provides energy (Oghbaei and Prakash, 2016). Carbohydrates in millets range from 64%-79% with sorghum having 72.6% carbohydrates (Gopalan et al., 1996). Major fractions of carbohydrates consist of amylopectin (72%-84%) and amylose (16%-28%). Among the free sugars *i.e.* glucose, fructose, raffinose, maltose and sucrose, sucrose is the major sugar present in millets, ranging from 0.3% to 1.2%, while the others range from 1% to 1.4% (**Table 4**). An approximate of 1.4-2% total sugars is found in small millets (Rao et al., 2018).

**Table 4:** Macronutrient profiling in millets as compared to cereals (per 100g).

Cereals and Millets	Carbohydrates (g)	Proteins(g)	Lipids(g)	Dietary fibers(g)	Moisture(g)
Wheat	64.72 ± 1.74	10.36 ± 0.29	1.47 ± 0.05	02.76 ± 0.29	10.58 ± 1.11
Rice	74.80 ± 0.85	09.16 ± 0.75	1.24 ± 0.08	04.43 ± 0.54	09.33 ± 0.39
Maize	64.77 ± 1.58	08.80 ± 0.49	3.77 ± 0.48	12.24 ± 0.93	09.26 ± 0.55
Bajra	61.78 ± 0.85	10.96 ± 0.26	5.43 ± 0.64	11.49 ± 0.62	08.97 ± 0.60
Ragi	66.82 ± 0.73	07.16 ± 0.63	1.92 ± 0.14	11.18 ± 1.14	10.89 ± 0.61
Sorghum	67.68 ± 1.03	09.97 ± 0.43	1.73 ± 0.31	10.22± 0.49	09.01 ± 0.77
Little millet	65.55 ± 1.29	08.92 ± 1.09	2.55± 0.13	06.39 ± 0.60	14.23 ± 0.45
Foxtail millet	60.09	12.30	4.30	–	11.20
Proso millet	70.04	12.50	1.10	–	11.90
Barnyard millet	65.55	06.20	2.20	–	11.90
Kodomillet	66.19 ± 1.19	08.92 ± 1.09	2.55 ± 0.13	06.39 ± 0.60	14.23 ± 0.45

(Source: Rao et al., 2018)

### 4.2 Proteins

Millets contain significant amounts of protein, which are of good quality with essential amino acids (Saleh et al., 2013). Protein content in millets varies from 7.7% to 11.8% (Rao et al., 2018) (**Table 4**). The three important fractions of protein present in millets are: i) globulin and albumin ii) prolamin like and true prolamin and iii) glutelin like and true glutelin of which glutelin comprises the highest fraction (45-55%) of total protein followed by prolamin (15%-30%) and albumin and globulin (8.5%-16.26%) (Rao et al., 2018). Millets are rich in sulphur containing amino acids like cysteine and methionine especially methionine which is generally deficient in cereals. Like in most cereals, millets are poor in tryptophan and lysine contents. Leucine (12-22.3%) and glutamic acid (16-23%) make up the prolamin fraction of protein in millets. Pearl millet has globulins plus albumins of 18%-26%, prolamins of 33%-49.5% and glutelins of 30%-45% (Rani et al., 2018).

### 4.3 Lipids

Millets generally have low-fat content, but the fats they contain are mostly unsaturated and considered healthy. In millets, most lipids, as indicated in **Table 4**, are primarily located in the pericarp, aleurone layers, and germ of the grain. Essential fatty acids are found in both free and bound forms. Free forms of fatty acids such as oleic acid, palmitic acid, and linoleic acid constitute approximately 60%-70% of the total, while bound forms include compounds like monogalactosul, diacylglycerols, phosphatidylethanolamine, digalactosyl diacylglycerols, phosphatidylcholine, and phosphatidylserine (Bagdi et al., 2011; Sridhar and Lakshminarayana, 1992). Fatty acids like erucic acid, arachidic acid, behenic acid are also found in millets but in lesser amounts. More than 85% of unsaturated fatty acids is present in millets (Annor et al., 2015). Linoleic acid (38%-40%), palmitic acid (16%-22%), oleic acid (27%-37%) and linolenic acid (1%-4%) are major fatty acids found in millets (Annor et al., 2015). Free lipid contents in various millets ranged from 3.4%-5% (Rao et al., 2018).

### 4.4 Dietary fibers

Millets are rich in dietary fiber, which aids in digestion and helps maintain gut health. Millets have higher content of dietary fibers as compared to other cereals (**Table 4**). Dietary fibers are of two types; insoluble dietary fibre (IDF) and soluble dietary fiber (SDF). Various studies suggest that IDF and SDF ranged from 1.5%-3% and 0.3-0.9% respectively (Rao et al., 2018). The dietary fiber content in millets fluctuates based on factors such as the variety, type of millet, and the process of decortication. For instance, in prosomillet, the dietary fiber content ranged from 12% to 20% prior to decortication, which then decreased to 3% to 5% after the process (Bagdi et al., 2011). Similarly, various types of finger millet are reported to contain dietary fiber in the range of 7% to 21.2% (Premavalli et al., 2004).

### 4.5 Mineral content

Millets serve as abundant sources of micronutrients, typically ranging from 1.7 to 4.3 mg per 100 grams, significantly surpassing the levels found in wheat (1.5%) and rice (0.6%) (Kumar et al., 2021). They contain substantial quantities of essential minerals such as iron, zinc, calcium, magnesium, and potassium, particularly when consumed as whole grains (Rao et al., 2018). Foxtail millet stands out for its high zinc content, with approximately 4.1 mg per 100 grams (Chandel et al., 2014), while finger millet offers substantial calcium, ranging from 300 to 400 mg per 100 grams (Rao et al., 2018). Additionally, sorghum is notably rich in potassium. These micronutrients play critical roles as coenzymes, supporting bone development, nerve conduction systems, muscular contractions, and various other vital functions within the human body.

### 4.6 Vitamins

Millets contain various vitamins, each contributing to their nutritional value. Millets are particularly rich in B vitamins such as niacin (B_3_), thiamine (B_1_), riboflavin (B_2_), and vitamin B_6_ (Kumar et al., 2021). Millets also contain vitamin E, which acts as an antioxidant, protecting cells from damage caused by free radicals (Santra and Rose, 2013). Niacin content is found to be highest in pearl millet among other cereals. B complex vitamins are found in relatively higher amount in sorghum than other cereals. Kodomillet is a good source of niacin, folic acid and B complex vitamins.

### 4.7 Carotenoids

The average values of carotenoids in various millets range from 1.99 mg/kg to 3.66 mg/kg, showing variability across different types (Asharani et al., 2010). The predominant components of carotenoids present in millets are β-carotene, cryptoxanthin, zeaxanthin and trans- or cis- luteine (Abdel-Aal et al., 2007). In prosomillet, lutein (5-15 mg/kg) and zeaxanthin (16-17 mg/kg) are major component of carotenoids (Zhang et al., 2014) whereas β-carotene is a minor component in foxtail, pearl and finger millets (Mamatha et al., 2011).

### 4.8 Phytochemicals

Phytochemicals are naturally produced plant compounds that don’t have an important role as nutrients but are very crucial for secondary metabolic activities in plants like providing resistance against any kind of biotic and abiotic stresses (Leitzmann, 2016). They are secreted by special type of cells in plants and occur in low concentration. Consumption of these have higher health advantage in humans because of its antiviral, anticarcinogenic, antimutagenic, anti-inflammatory and anti-oestrogenic properties (Kumar et al., 2021). These include compounds like phenolic acid, lignans, anthocyanins, β-glucans, phytosterols, tocopherols, flavonoids etc. which are quite abundant in millets. Polyphenols are complex mixture of benzoic acid and cinnamic acid derivatives most of which are found in seed coat of millets, which are found to be reduced after milling (Kaur et al., 2012). Generally, catechin/epicatechin, apigenin, glycoside, kaempferol, phloroglucinol, catechin gallates, trans-feruloyl-malic acid and daidzein are the polyphenolic compounds that are found to be present in the seed coat of millets (Liu et al., 2012; Nakarani et al., 2021). Among the common flavonoids present in millets’ leaves are orientin, isoorientin, violanthin, vitexin, isovitexin, violanthin, saponarin, tricin etc. Millets exhibit higher antioxidant activities due to presence of flavonoids, condensed tannins, and polyphenols (Kumar et al., 2021) compared to cereals and fruits.

### 4.9 Antinutrients

Millets contain various antioxidants such as phenolic compounds and flavonoids, which have potential health benefits. Compounds like phytates, tannins, oxalates and phenols act as antinutrients due to their metal chelating and enzyme inhibitory nature. These compounds generally form complexes with mineral ions of zinc, calcium, iron etc. rendering them unavailable biologically to human beings (Arora et al., 2003). Some millet processing methods like soaking, decortication, fermentation, malting, cooking etc. reduce the concentrations of antinutrients, which make millets more digestible and the minerals more available to human beings.

## 5 Nutritional and health benefits of different millets

Though all the millets are known for their health benefits, the nutritional content and health benefits provided by them tend to vary due to the presence of differing concentrations of nutrients such as carbohydrates, proteins, fats, dietary fibers, vitamins, minerals, etc. Millets come in a variety of shapes, sizes, and colors as shown in **Figure 1**. Nutritional and health benefits of different millets are discussed below:

### 5.1 Foxtail millet

Foxtail is a highly nutritious millet, which contains proteins, fiber, essential amino acids, fatty acids, vitamins, and minerals. Phenolics and flavonoids are also present which act as antioxidants (Abedin et al., 2022). They are low glycemic index foods and possess certain compounds such as alpha glucan, which helps in faster sugar and cholesterol metabolism making them ideal food for diabetes patients and cardiovascular diseases (Ramashia et al., 2019). In addition, foxtail millet has anti-cancer properties (Zhang and Liu, 2015).

### 5.2 Kodo millet

Loaded with high protein, carbohydrates, minerals, crude fibers, polyphenols, and antioxidants, kodo millets have numerous nutritional and health benefits. The extract of kodo millet contains high polyphenols, which showed very good antibacterial activity (Sharma et al., 2017). Moreover, they are anti-diabetic, anti-mutagenic, and anti-carcinogenic (Ferguson, 2001; Shobana et al., 2007; Vetriventhan and Upadhyaya, 2019). Consumption of kodo millet is good for post-menopausal women suffering from cardiovascular diseases and high cholesterol levels (Hedge et al., 2005; Rao et al., 2016).

### 5.3 Proso millet

Proso millet is rich in protein, dietary fiber, vitamins, minerals, phytochemicals, and micronutrients including iron, zinc, copper, and manganese. Thus, its incorporation in the daily food diet may overcome nutrient deficiencies. It contains a significant amount of lecithin which is important for the overall neural health system. They are effective in preventing certain forms of cancers such as colon cancer, hormone dependent cancer, breast cancer, and liver cancer (Zhang et al., 2014; Coulibaly et al., 2011).

### 5.4 Finger millet

Finger millet is a rich source of protein, dietary fiber, phytochemicals, vitamins, and minerals such as calcium, iron, and potassium. Being a fantastic source of calcium among all the millets, they are known to strengthen bones and alleviate osteoporosis symptoms by restoring optimum bone density in older people. Many studies have shown anti-diabetic and anti-inflammatory effects of finger millet due to its dietary fiber and phenolic compounds (Kumar et al., 2016; Chaudhary and Mudgal, 2020; Singh et al., 2022). It has massive benefits for pregnant and lactating women to stimulate milk production, regulate hormonal balances and prevent anemia due to the presence of calcium and iron. Finger millet may tackle lifestyle disorders, chronic and non-communicable diseases (Nakarani et al., 2021) and boost nervous system function due to elevated levels of amino acid tryptophan.

### 5.5 Pearl millet

Pearl millets are energy- and protein-rich foods that are comparable with other cereals such as rice, wheat, maize, and sorghum. They are also rich in B vitamins and minerals such as potassium, phosphorus, magnesium, zinc, iron, manganese, and copper. The presence of high fiber contents can help tackle health problems such as obesity, constipation, and rich omega-6 fatty acid content for lowering the risk of cardiovascular disease (Kumar et al., 2020; Baltensperger, 2002). The phytates and polyphenols present in pearl millets assist in controlling aging and metabolic diseases. In addition, the phenolic compounds present mainly in the pearl millets such as p-coumaric and ferulic acids are known to have anti-cancer properties (Chandrasekara and Shahidi, 2011).

### 5.6 Little millet

Little millets are rich in protein, fiber, magnesium, zinc, phosphorous, iron, and high amount of vitamin B. They are recommended for people with diabetes, high blood pressure, high cholesterol and polycystic ovary syndrome (PCOS) etc. The high antioxidant content in them makes a good diet to fight toxins and build immunity for overall health. Little millet contains various bioactive compounds such as apigenin, which is anti-diabetic as well as anti-cancerous (Thakur and Tiwari, 2019); luteolin which is antioxidant and anti-inflammatory (Dykes and Rooney, 2006); Kaempferol for lowering the risk of chronic disease (Pradeep and Sreerama, 2015).

### 5.7 Barnyard millet

Apart from being delicious, barnyard millet is rich source of protein, iron, dietary fiber, phosphorus and is low carbohydrate which makes it effective in controlling the blood sugar level, blood pressure, and the cholesterol level that keeps the heart healthy; it also prevents from celiac diseases due to the absence of gluten. They have anti-diabetic properties because of their low glycemic index and the presence of polyphenol compounds, which help reduce the breakdown of complex sugars and prevent a spike in blood sugar levels. They are known to fight anemia and help manage gastrointestinal disorders such as constipation, bloating and acidity due to the presence of iron and fiber, respectively. Vanillin compound extracted from barnyard millet may reduce the risk of cancer especially breast cancer (Ramadoss and Sivalingam, 2021).

### 5.8 Sorghum

Sorghum acts as an excellent source of protein, dietary fiber, vitamins (mainly B), and minerals like calcium, iron, potassium, copper, phosphorus, sodium, and zinc. It is a great choice for people with obesity and digestive problems due to its high concentration of dietary fiber. The presence of essential minerals such as iron and copper boosts blood circulation and prevents anemia by improving the development of red blood cells. Sorghum exhibits anti-diabetic properties due to its complex carbohydrates, which are digested slowly, helping to maintain stable blood sugar levels. In addition, it has anti-carcinogenic effects and may help lower the risk of esophageal cancer (Dykes and Rooney, 2006).

## 6 Important food and non-food products from millets

**Figure 3:**
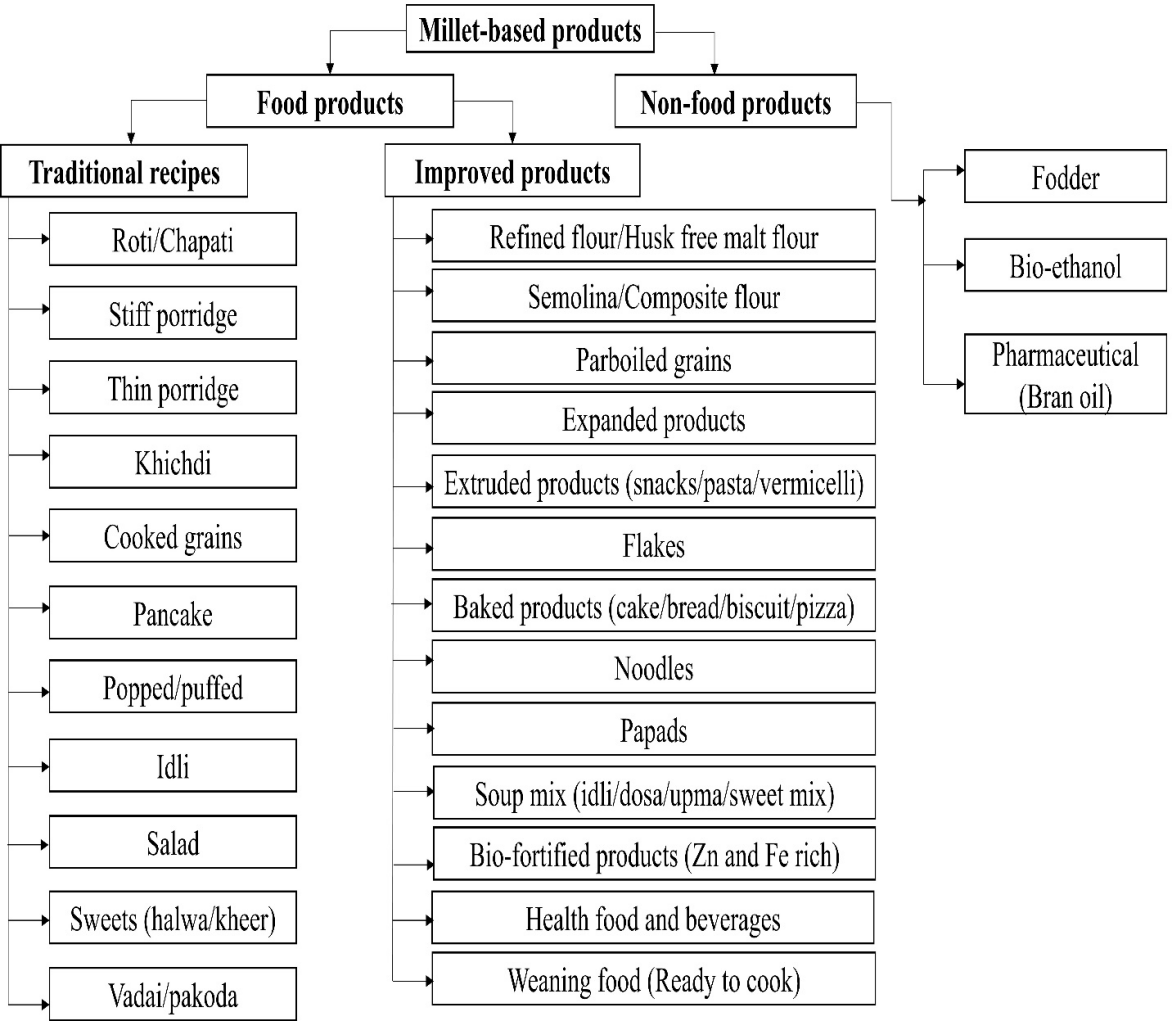
Millet based products developed through different processing technologies (Ref: Rao et al., 2016; Shi et al., 2015; Yemets et al., 2020).

Traditionally, processing technologies such as dehusking, milling, malting/germination, roasting, popping and cooking were practiced for preparation of various traditional recipes. The continuous search for nutritive alternative due to consumer’s awareness and demand has led the incorporation of millets in food products which have become an emerging trend for Food industries (Knowledge paper on Millets -The Future Super Food For India, 2022). In Indian context, ICAR-IIMR (Indian Institute of Millets Research) has made attempts to innovate technologies that enable the development of sorghum/millets-based value-added products through different projects. **Figure 3** shows the different millet-based products developed through different processing technologies. Moreover, non-food products such as bioethanol and bran oil from the residues of millet have been successfully produced.

## 7 Role of millets as climate smart crop

Despite all the evidence to the contrary, climate change is an undeniable fact of our time and is being felt all over the world through changes in rainfall pattern, glacier melting, and rising air temperature etc (Knappenberger et al., 2001). Under such climate changing scenario, the productivity of major cereal crops will decline drastically, and it may pose a huge threat to the burgeoning population around the globe. In developed countries, the production of many cereal crops has stagnated, while the overconsumption of rice, wheat, and maize has led to a rising epidemic of serious health problems, such as diabetes, cancer, cardiovascular diseases, obesity, malnutrition, and overeating (Yang et al., 2011). So, to combat this situation, scientists and researchers need to formulate climate smart agricultural strategies to ensure food for global population.

In light of climate change, millets are an alternate crop. They can withstand the negative effects of climate change and adjust to the altered and broader agro-climatic circumstances. Obviously, they are known as climate-smart crops (Bandyopadhyay et al., 2017; Yadav et al., 2013). Millets are more resilient to environmental stressors than major cereals due to a number of morpho-physiological, molecular, and biochemical traits. Since millets only need 12–14 weeks to complete their life cycle (from seed to seed), compared to a maximum of 20–24 weeks for rice and wheat, their short life cycle primarily helps them avoid lots of biotic and abiotic stresses (Bandyopadhyay et al., 2017). Millets can be an effective alternative for such climate changing scenario because of their ability to adapt to wide range of weather conditions, low irrigation requirement, better capability to produce grains under nutrient-poor soil and least vulnerability to biotic and abiotic stresses (Kole et al., 2015; Scopa et al., 2023). Millets’ short stature, tiny leaf area, thickened cell walls, and capacity to establish dense root systems are some of the characteristics that help them avoid the prevalence of stress conditions and their effects (Li and Brutnell, 2011). Being low water consuming crop (200-250 mm), the rainfall needed for sorghum, pearl millets and finger millet is 25% less than sugarcane and banana, and 30% less than rice (MINI, 2023).

Most of the millets are short-duration cereals as they can complete their life cycle within 84-98 days which is very earlier compared to other cereals like rice and wheat etc (Paschapur et al., 2021). Apart from providing water use efficiency (WUE) and nutrient use efficiency (NUE), C_4_ photosynthesis offers millets additional advantages such as better growth and ecological performance in warm climates, more adaptable biomass allocation patterns, and lower hydraulic conductivity per unit leaf area (Sage and Zhu, 2011). Several case studies have also shown the climate resilient nature of millets. For example, pearl millet adjusts flowering phenology according to rainfall pattern as per the experiment conducted by Bidinger et al. (2007). Ajithkumar and Panneerselvam (2014) reported an increase in root length in little millet during drought conditions. They also showed enhanced levels of reactive oxygen species, antioxidants and stress combating enzyme (catalase and superoxide dismutase) activity during abiotic stress situations. Millets have a substantially smaller carbon footprint than other crops, which can reduce the global carbon impact also (Prasad and Staggenborg, 2009).

**Figure 4:**
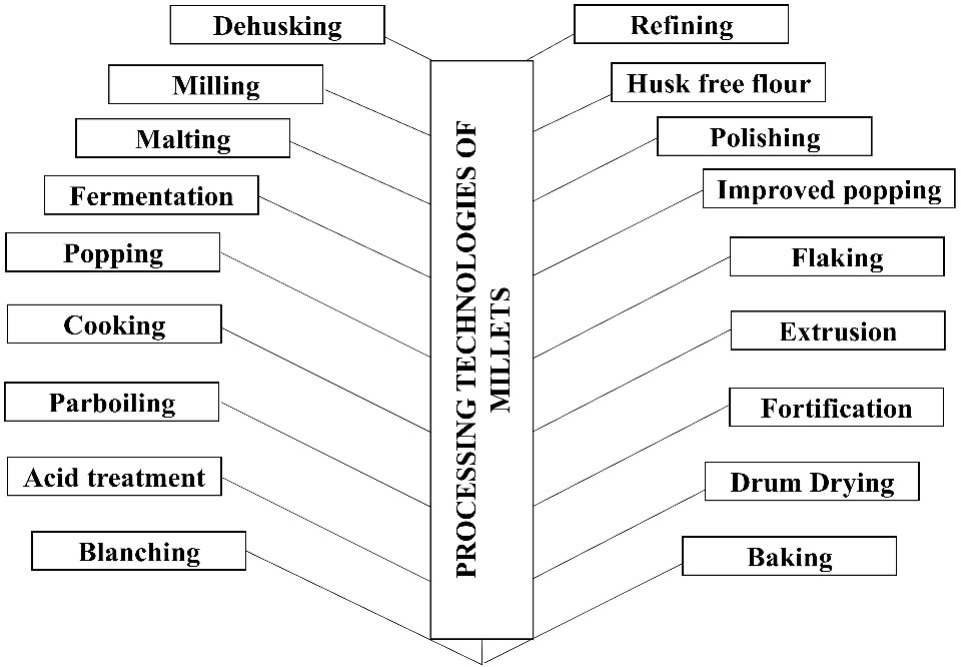
Processing technologies of millets (Ref: Rao et al., 2016; Yousaf et al., 2021; Selladurai et al., 2023) .

Millets are considered underutilized cereals despite having a vast potential source of nutrition and health benefits. The decline in direct consumption of such nutritious grains might be attributed to their restricted consumption only in limited rural growing areas, difficulty in food preparation as compared with other cereals, lack of awareness of their health benefits, lack of processing technologies, and lack of incentives from the Government side. However, with the growing concern for nutritive intake of food over the past decade, researchers and food scientists are developing technology to make millet-based value added products available as convenient to prepare and easily accessible at reasonable prices in urban market (**Figure 4**).

## 8 Advances in millets research: genetics and genomics

Millet improvement efforts rely heavily on the exploration of genetic and genomic resources (Singh and Upadhyaya, 2015; Dhaka et al., 2021). Various techniques such as molecular markers, functional genomic analysis, genome-wide association mapping, genomic-assisted breeding, and biotechnological advancements play key roles in this endeavour (Das et al., 2023; Dutta et al., 2023; Choudhary et al., 2023). The focus is on enhancing population diversity and maximizing the potential of donor genotypes for crop improvement. Pearl millet holds the largest share of germplasm collections at ICRISAT, followed by other millet varieties (Upadhyaya et al., 2011 & Upadhyaya et al., 2017). Genetic diversity can be assessed using molecular markers like single nucleotide polymorphisms and simple sequence repeats (Dhaka et al., 2021; Muthamilarasan and Prasad, 2021). Draft genome sequences of several millet species are available, aiding in genetic assessment. Foxtail millet, in particular serves as a model plant for stress tolerance and biofuel traits due to its genetic characteristics and accessible genomic resources (Bennetzen et al., 2012; Varshney et al., 2017; Hittalmani et al., 2017; Zou et al., 2019). Several concerted efforts have been made in genomic research on small millets, including the development of draft genome sequences, application of molecular markers, genome-wide association studies, and the use of foxtail millet as a model system for understanding stress tolerance and biofuel-related traits (Dutta et al., 2021).

Finger millet (*Eleusine coracana* (L.) Gaertn subsp. *coracana*) is an allotetraploid (2n = 4x = 36, AABB) with a genome size of approximately 1.45–1.59 Gb (Hittalmani et al., 2017; Hatakeyama et al., 2018; Devos et al., 2023). It likely originated from hybridization between *E. indica* (AA) and a B-genome donor. Domesticated over 5,000 years ago in eastern Africa, it shows rich genetic diversity, with over 37,000 accessions conserved globally (Kasule et al., 2024; Tiwari et al., 2020). The largest collection is maintained by India’s NBPGR, while ICRISAT also houses over 7,500 accessions from 26 countries. Initially reliant on morphological markers, breeding efforts have since incorporated molecular tools such as RAPDs, SSRs, and SNPs (Gimode et al., 2016; Joshi et al., 2021; Kasule et al., 2023). The adoption of next-generation sequencing enabled comprehensive diversity assessments using platforms like GBS (Brhane et al., 2022) and DArTseq (Backiyalakshmi et al., 2021). GWAS has revealed key marker-trait associations for yield, nutrition, and disease resistance. Despite these advances, finger millet lacks a centralized genomic database, limiting the efficiency of molecular breeding compared to crops like sorghum. Establishing such a platform could integrate genomic, QTL mapping, and phenotypic data integration to accelerate breeding and gene discovery (Roy et al., 2024). Further sequencing, especially from regions of origin and wild relatives, would enrich marker density and reveal traits for resilience. A pan-genome capturing the crop’s genetic diversity is urgently needed to support gene pyramiding and genome editing for durable resistance and stress tolerance. Investing in such genomic infrastructure and leveraging CRISPR/Cas9 will be crucial to unlocking finger millet’s potential for sustainable agriculture in the face of climate change (Dutta et al., 2024; Kasule et al., 2024; Goron and Raizada, 2015).

Proso millet (*Panicum miliaceum* L.) is a self-pollinated tetraploid species (2n = 4x = 36) with a genome size of approximately 923 Mb (Zou et al., 2019). It is an ancient cereal crop, domesticated over 10,000 years ago and primarily cultivated in northern China. Though proso millet is largely self-pollinating, natural outcrossing occurs at a low rate (<10%) (Kasule et al., 2024; Boukail et al., 2021). It is widely grown in countries like China, the USA, India, Nepal, and several Eastern European and African nations. Globally, about 29,308 proso millet accessions are conserved in gene banks (Kasule et al., 2024). Major repositories include the Vavilov Institute (8778 accessions), ICGR-CAAS (6517), and ICRISAT (849 accessions from over 30 countries). Genetic diversity has been assessed using a range of molecular markers such as AFLP, RAPD, SSR, SRAP, and SNPs (Kasule et al., 2024). The draft genome sequences of proso millet include ‘Pm0390’ (923 Mb, 55,930 genes) and ‘Longmi4’ (887.8 Mb, 63,671 genes). A graph-based pan-genome, constructed from 32 accessions and short-read data from 516 lines, revealed 50,689 structural variants and enabled the identification of 139 loci associated with 31 traits, including seed color and shattering (Chen et al., 2023). Despite these advances, proso millet lacks a centralized genomic database, which limits research and breeding efforts. Dedicated platforms could offer curated data, powerful analytical tools, and collaboration opportunities. Looking forward, integration of the pan-genome and CRISPR-based editing could unlock genetic improvements for yield, stress tolerance, and nutrition. Establishing comprehensive databases and optimized transformation protocols will be vital for leveraging genomic insights to develop resilient, high-yielding proso millet cultivars, thereby enhancing its role in global food security (Yadav and Rai, 2013).

Foxtail millet (*Setaria italica*), a diploid annual plant with 2n = 2x = 18 chromosomes, was domesticated from *Setaria viridis* around 8,000–10,000 years ago in China (Diao and Jia, 2017; Bettinger et al., 2010; Lata et al., 2013). As a self-pollinating crop (0.3–4% outcrossing) with a compact genome (~499–509 Mb), it has emerged as a model for C4 grasses and is extensively cultivated in arid and semi-arid regions of 23 countries for food, feed, and fodder (Kheya et al., 2023). A vast germplasm collection exceeding 46,000 accessions exists globally, with major holdings in China, India, France, Japan, the USA, and Kenya. Core and mini-core collections have been evaluated for traits like maturity, plant height, grain yield, nutrition (Fe, Zn, Ca), and stress responses (Kasule et al., 2024). Studies using molecular markers such as SSRs, SNPs, and ISSRs highlight the rich genetic diversity of foxtail millet (Kasule et al., 2024). Although early genomic progress was limited, advancements in sequencing technologies have accelerated research. Recent pan-genome analyses of 75 foxtail millet assemblies revealed extensive structural variants and gene families (Kasule et al., 2024). A graph-based genome integrating these variants enabled genotyping of 1,844 accessions, uncovering over 60 million SNPs and 6.7 million indels (He et al., 2023). GWAS across 68 traits identified key loci for adaptation and yield traits. Dedicated databases like FmMDb, FmTFDb, and MDSi support marker development, transcription factor analysis, and SNP tracking (Kasule et al., 2024). To fully harness foxtail millet’s potential, further inclusion of accessions from Africa, Europe, and the Americas is crucial. Expanding GWAS to address biotic and abiotic stress traits will enhance climate resilience, promote genetic gains, and strengthen global food security.

Successful genetic transformation systems have been developed for various millet species, facilitating transgenic research (Singh and Prasad, 2016; Sood et al., 2019; Mundada et al., 2022). Additionally, virus-induced gene silencing methods enable functional gene evaluation in millets and other cereal crops (Bouton et al., 2018; Liu et al., 2016; Mei et al., 2016). These advancements are crucial for accelerating millet breeding and crop improvement efforts. Cutting-edge omics and breeding techniques have unveiled significant nutritional and climate-resilient features in millets, accelerating millet development programs (Chaudhuri et al., 2023). Genomic technologies play a pivotal role in identifying trait-responsive genes for breeding. Whole genome sequence data for tiny millets like foxtail millet, finger millet, barnyard millet, teff, and green foxtail have expedited the identification of novel genes controlling nutritional and climate resilience features (Hittalmani et al., 2017; Bennetzen et al., 2012; Guo et al., 2017; Muthamilarasan et al., 2019; VanBuren et al., 2020; Muthamilarasan and Prasad, 2021). Genome-wide association studies (GWAS) have identified loci related to various dietary components and agronomic traits (Jaiswal et al., 2019). Omics methodologies such as transcriptomics, proteomics, and metabolomics enable the quantitative and qualitative examination of candidate genes and regulatory networks (Dhaka et al., 2021). RNA sequencing studies have revealed salt and drought-responsive genes in small millets, providing candidates for crop improvement (Varshney et al., 2017; Das et al., 2020; Dutta et al., 2020). Proteomics research has identified proteins responding to drought in millets. Integration of omics data facilitates the identification of candidate genes for gene-editing methods. Genomic research has also uncovered markers linked to disease resistance in small millets, aiding in the screening of resistant germplasms (Choudhary et al., 2023; Dutta et al., 2023; M'Ribu and Hilu, 1996). The advancement of phenomics and computational methods further accelerates crop improvement programs for small millets (Pan et al., 2018). Thus, biotechnology and computational methods hold promise in enhancing millet breeding and resilience to climate change.

## 9 Agronomic interventions for upscaling millet cultivation

Despite having immense agricultural value, the area under millet cultivation and production have declined compared to major cereals. Nonetheless, the advent of high yielding cultivars and enhanced production technologies have increased the productivity of these crops. Research on more productive and economical crop management techniques is essential to boost their output and predicting their future as crops. Location specific adaptive research on crop management has led to development of improved practices for sowing, nutrient management, weed management, irrigation, farm mechanization and cropping systems suitable for different millet growing states. Some important agronomic interventions related to millet cultivation which are being followed by researchers for improving the productivity are as follows:

### 9.1 Sowing window

Millets are warm-weather crops that are physiologically efficient and are part of the C4 group of plants. Proper sowing time is one of the important non-monetary inputs for efficient crop production as it provides appropriate environment at all the growth stages. The results of various field experiments conducted across the country revealed that the first and second fortnight of July are the most suitable period for cultivating different types of millet. The results of an experiment carried out in Bengaluru, Nandyal, and Waghai between 2016 and 2019 showed that the best times to sow foxtail millet, small millet, and proso millet were during July 1^st^ and 2^nd^ fortnight of each week. However, planting beyond the 2^nd^ fortnight of July led to a sharp decline in crop production (Sukanya et al., 2022). As per field experiment on pearl millet conducted at Peshawar, Pakistan, crop sown on 20th June through transplanted method had maximum panicle weight, grains panicle^-1^, 1000 grains weight and grain yield (Jan et al., 2015).

### 9.2 Nutrient management

Crop production depletes soil nutrient supplies if nutrients are not managed properly. A greater yield of agricultural products depletes the soil of vital nutrients in significant quantities. The productivity of agricultural systems based on millet has increased through the integrated use of fertilizers and organic materials. Numerous studies on millets have unequivocally shown that INM techniques are more popular and generally more successful in millet crops (Sukanya et al., 2022). Usage of bio-fertilizers like *Azospirillium* and PSB with recommended dose of fertilizer, combined application of organics with NPK, foliar nutrition of micronutrients, SSNM or precision nutrient management approaches have been proven beneficial for productivity enhancement in millets (Louhar et al., 2020).

### 9.3 Alteration in intercropping system

The term “cropping system” refers to all agricultural cropping patterns and how they interact with other household businesses, farm resources, and environmental, biological, technological, and sociological elements. Small millets benefit greatly from intercropping because it makes better use of growth resources like light, nutrients, and water; suppresses weeds; increases yield stability; increases equivalent yields (yield of base crop plus yield of intercrop) and cropping intensity; lowers incidence of pests and diseases; and improves soil health and agro-eco system (Sukanya et al., 2022). Small millet-based pulse intercropping system like intercropping of finger millet and foxtail millet with red gram, little millet with black gram or sesame, kodo millet with sesame has been proven to be beneficial across different AICRP centers on Millet across the country (Sukanya et al., 2022).

### 9.4 Crop rotation

Several experiments also proved that following proper crop rotation across different locations of the country can be very beneficial for millets productivity. The results from Bangalore during the kharif seasons of 1998 and 2001, in which four crops—finger millet, groundnut, sunflower, and maize were subjected to four levels of nutrition and the recommended dose of FYM (7.5 t/ha), showed that the finger millet-groundnut crop rotation ranked highest in terms of gross monetary returns, followed by the finger millet-maize rotation (Sukanya et al., 2022).

### 9.5 Weed management

Like in other cereals, different types of weeds like *Parthenium hysterophorus*, *Cynodon dactylon*, *Alternanthera phyloxiroides*, *Eleusine indica*, *Portulaca oleracea* etc. are found in different millet growing field. Studies on weed control in millets have been initiated since the inception of AICRP on Small millets, and effective weed management has been considered as important operation in millet farming (Sukanya et al., 2022). Keeping the plots weed free up to 30-40 days from sowing was found ideal and effective in improving millet productivity. The study conducted in 2001 at the centers in Bangalore, Bengaluru, Coimbatore, Jagadalpur, and Ranchi showed isoproturon @ 0.5 kg a.i./ha as pre-emergence spraying was more advantageous, and that combining herbicide treatment with mechanical and cultural weed control measures can further improve weed control while also increasing yield (Sukanya et al., 2022).

Further, more agronomic operations like sowing with seed drill, intercultivation, mechanical harvesting, conservation and response farming etc. are also practiced across different locations of the country for ensuring better productivity of millets.

## 10 Challenges and prospects of millet cultivation

Although millets are climate-smart crops, they have lost their traditional prominence in the mainstream food market due to policies that have prioritized the promotion of crops like wheat and rice, particularly following the Green Revolution. Lack of proper awareness about millets fulfilling nutritional security and combating climatic variability has led to poor research, marketing, and value-added processing industry for almost seven decades. The main challenges that are being faced for the prolific commercialization of millets are:

1. Presence of a weak supply chain that connects growers to processors including intermediaries that increases the cost component by 40% (Lokesh et al., 2022).
2. Customer awareness regarding the nutritional benefits of millets is inadequate since most of the information is informal and word-of-mouth.
3. Millets are not popular among farmers because of its lower yields of about 4-5 q/ha as compared to main cereals like rice, wheat, and maize having yields of more than 20-25 q/ha (Lokesh et al., 2022).
4. Due to lack of proper processing machineries, recovery percentage is 60-65% for which many by-products of millets are left unexplored leading to higher selling prices.
5. There is a dearth of suitable processing technologies resulting in unavailability of desirable value-added millet products in both domestic and international markets.
6. Absence of government policies to improve production and market chain logistics.
7. Lack of standard protocols for production, processing, and marketing of millets.
8. Non-inclusion of millets in Indian public distribution systems (PDS).

**Figure 5:**
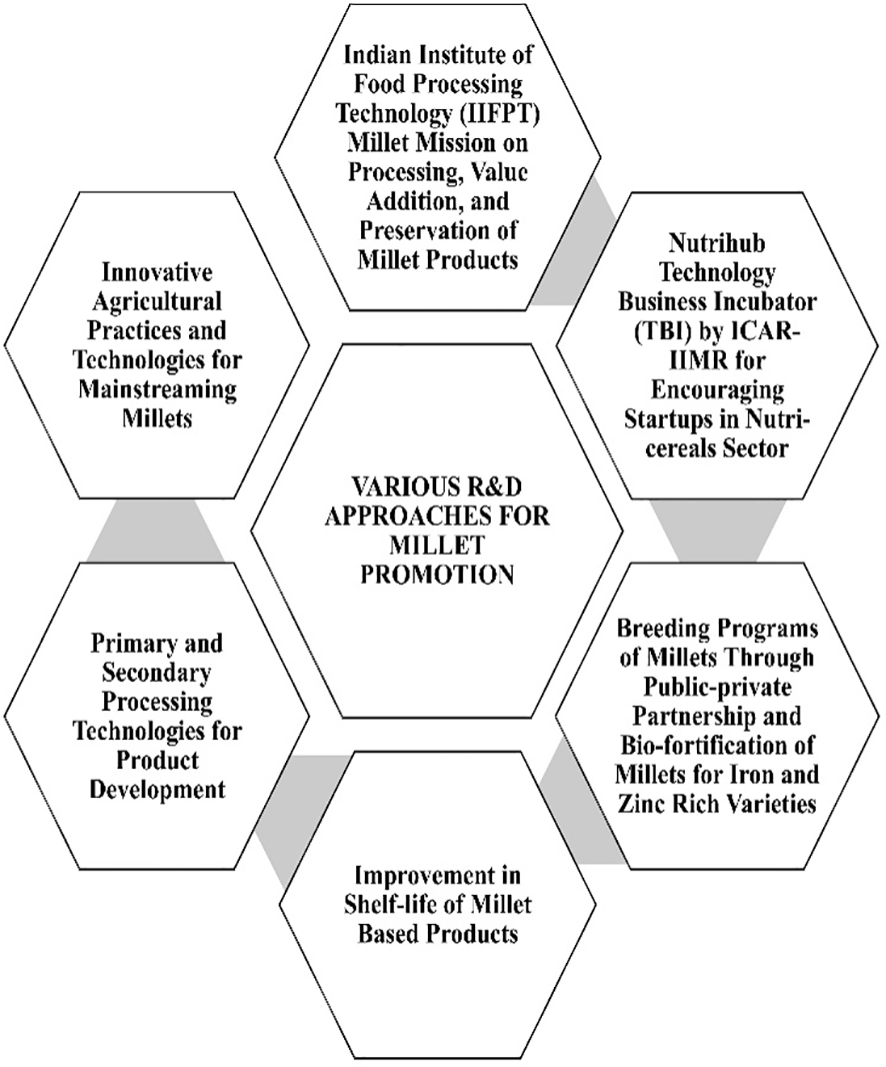
Schematic representation of research and development components for millet production.

Overcoming all these challenges is crucial for commercialization of millets. Continuous research on new production technologies for millets and proper government initiatives are essential to achieve the said objectives of making millets popular among producers as well as customers (**Figure 5**). Some of the interventions already implemented and those to be popularized are given below:

1. More schemes like INSIMP (Initiative for Nutritional Security through Intensive Millets Promotion) should be launched nationwide, where farmers would be getting input support and services in the form of free certified seed minikits, training, and incentive for hybrid seed production. Schemes related to improved post-harvest processing technologies and awareness among consumers should be promoted more (The Associated Chambers of Commerce and Industry of India, 2022).
2. INSIMP was continued as NFSM (National Food Security Mission) during 2012-2017, having NFSM-Coarse cereals as its important component. Inculcating improved package and practices of millets along with hybrid seed distribution has been its main motto making millets quite popular among consumers (Lokesh et al., 2022)
3. Insurance and marketing support should be extended to every millet producer.
4. New developed processing machineries are to be made available to millet farmers for better value-added products (The Associated Chambers of Commerce and Industry of India, 2022).
5. Exposures of millet producers to various international research organizations like ICRISAT, CIMMYT, ICARDA etc. should be funded by government so that they can imbibe advanced crop production technologies.
6. Inclusion of millets in the mid-day meal programme should be recommended nationwide. Already Karnataka has included millets in the mid-day meal programme (Lokesh et al., 2022).
7. Minor millets should be given MSP support just like major millets that were covered under MSP during 2021-2022.
8. Organic Millet Mission (OMM) has been successfully initiated by state of Orissa in India in which millets are being distributed by PDS system.
9. Andhra Pradesh has started millet board in 2020 in which they are disseminating awareness about benefits of millets along with building processing units.
10. Establishment of online millet stores as well as grocery stores need market boosts related to millets.
11. According to recent report published by NITI Aayog various states and UTs have taken up innovative practices to promote millets by including in State Missions (Sen et al., 2023).

## 11 Conclusion

**Figure 6:**
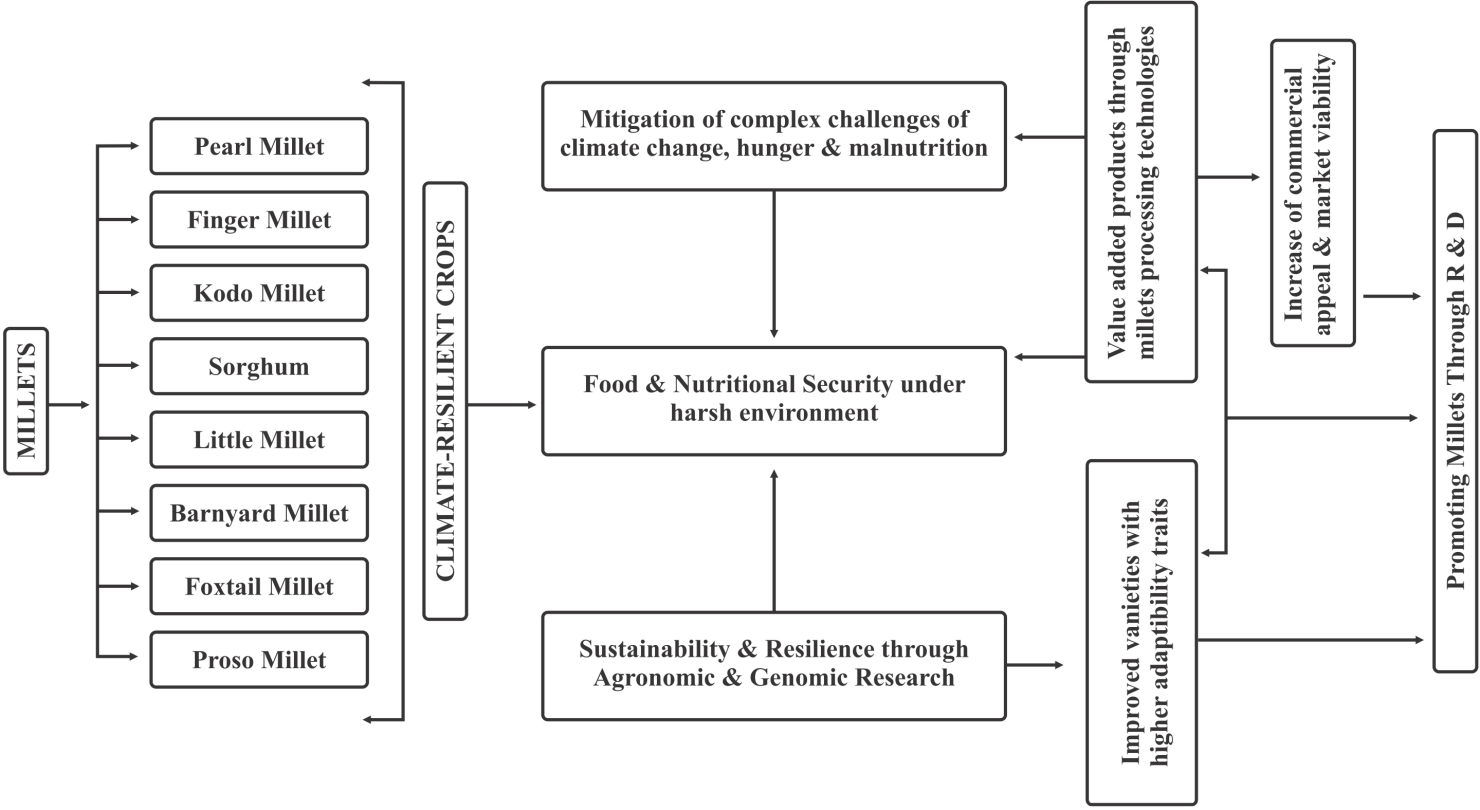
Schematic diagram depicting importance of millets research, processing and marketing.

Millets exhibit remarkable resilience to harsh environmental conditions such as drought, with some wild varieties even thriving in flooded fields and marshy environments. These contain gluten-free protein, low glycemic index carbohydrates, and a wealth of minerals, vitamins, and antioxidants. These exceptional qualities make them nutrient-dense and resistant to climate change crops. These can benefit the health of the community in addition to providing farmers with a source of revenue. Challenges such as the presence of antinutritional components and limited sensory acceptance of millet-based products must be addressed through scientific interventions. The summary image given below illustrates the outputs of millet research, processing, and marketing as well as the viability of millet marketing in the context of climate change (**Figure 6**).

Integrating millets into processed and packaged foods can incentivize farmers to cultivate them and unlock new economic opportunities. The current nutrient deficits of protein, calcium, and iron in poor nations can be addressed by the inclusion of millet-based foods in international, national, and state-level feeding programmes. The inherent climate resilience and superior nutritional profile of small millets position them as crucial components of sustainable food systems, surpassing traditional staples like rice and wheat. Therefore, crop diversity is crucial in the current environment to achieve food and nutritional security. The functional characterization of significant genes from small millets in other crops has been published in numerous previous research. Small millets, however, lack such attempts because there are not any standardized transformation techniques. A complete genome sequence of various small millets could be provided using modern sequencing techniques. Furthermore, cutting-edge phenomics techniques supported by machine learning may speed up the gathering of genetic diversity data. The agricultural community has intensified its focus on small millet improvement to attain food, nutrition, and economic security considering the current threatening climate conditions and rising hunger.
